# Digital Health and Telemedicine Interventions for Hypertension Management in Adults: A Systematic Review

**DOI:** 10.7759/cureus.97024

**Published:** 2025-11-16

**Authors:** Naama Raquib, Mohamed Alshaab M Alshibane, Nuha Hamid Mohammed Awad Elkarim, Amina Osman, Yara Ahmed, Fatima Mohammed Ahmed Elhaj, Israa Mohamed Ahmed Dafaalla Abbas

**Affiliations:** 1 Internal Medicine, Grange University Hospital, Newport, GBR; 2 Cardiology/Biomedical Science, University of Reading, Reading, GBR; 3 Cardiology, Heart Hospital, Hamad Medical Corporation, Doha, QAT; 4 Acute Medicine, Queen Elizabeth Hospital, Gateshead, GBR; 5 Medicine, Faculty of Medicine, University of Medical Sciences and Technology (UMST), Khartoum, SDN; 6 Internal Medicine, Gold Trust Medical Center, Ajman, ARE; 7 Medicine, Faculty of Medicine, Al Neelain University, Khartoum, SDN

**Keywords:** blood pressure, digital health, hypertension, mhealth, remote monitoring, systematic review, telemedicine

## Abstract

Hypertension is a major global health challenge, with many patients failing to achieve optimal blood pressure (BP) control. Digital health and telemedicine interventions offer a potential solution by enabling remote monitoring and personalized management. This systematic review aims to evaluate the effectiveness of these technologies in improving clinical and behavioral outcomes in adults with hypertension. This review was conducted following the Preferred Reporting Items for Systematic Reviews and Meta-Analyses (PRISMA) guidelines. A comprehensive search of multiple databases was performed for randomized controlled trials (RCTs) published between 2020 and 2025. Thirteen studies meeting the inclusion criteria were selected. Data on study characteristics, BP outcomes, medication adherence, and lifestyle changes were extracted. The Cochrane Risk of Bias tool was used for quality assessment, and a narrative synthesis was performed due to the heterogeneity of the interventions. The included studies demonstrated that digital interventions, such as smartphone applications, SMS messaging, and remote monitoring, led to significant reductions in systolic BP. Improvements in diastolic BP, BP control rates, and medication adherence were also observed. Furthermore, the interventions were associated with positive lifestyle modifications, including reduced sodium intake, increased physical activity, and enhanced self-management capabilities. The methodological quality of most studies was assessed as having a low risk of bias. Digital health and telemedicine interventions are effective tools for improving hypertension management. They contribute to significant BP reduction, better control rates, and positive behavioral changes. Future research should focus on long-term sustainability, comparative effectiveness, and strategies to ensure equitable access to these technologies.

## Introduction and background

Hypertension remains one of the most significant global public health challenges, contributing substantially to cardiovascular morbidity, mortality, and healthcare costs [1]. According to the World Health Organization (WHO), an estimated 1.4 billion adults worldwide are living with hypertension, with nearly half unaware of their condition and only one in five achieving adequate blood pressure (BP) control [2]. Despite the availability of effective antihypertensive medications and evidence-based management guidelines, adherence to treatment, self-monitoring, and regular follow-up remain suboptimal in many populations [3]. These persistent gaps highlight the need for innovative strategies to improve hypertension detection, management, and long-term control.

In recent years, digital health technologies, an umbrella term referring to the use of digital tools and platforms such as mobile phones, wearable sensors, and internet-based systems to deliver or support healthcare, have emerged as promising solutions to these challenges [4]. These technologies encompass mobile health (mHealth) applications, telemedicine, remote monitoring, and web-based communication platforms, all designed to complement traditional in-person care. The rapid advancement and accessibility of these tools have enabled patients and healthcare providers to interact beyond conventional clinical settings. Through real-time communication, remote BP monitoring, and personalized feedback, digital health interventions can facilitate lifestyle modification, medication adherence, and timely clinical decision-making [5].

Telemedicine, a key component of digital health, refers specifically to the remote delivery of healthcare services using telecommunications technologies such as video consultations or phone calls. It has become increasingly integrated into chronic disease management, particularly in response to the COVID-19 pandemic, which greatly accelerated its adoption worldwide [6].

Several digital approaches have been explored in hypertension management. Mobile applications and text message reminders have shown potential in promoting medication adherence and supporting behavioral changes such as dietary improvement and physical activity [7]. Remote BP monitoring devices allow patients to record and transmit their readings to clinicians, enhancing engagement and enabling data-driven treatment adjustments [8]. Additionally, artificial intelligence (AI)-based algorithms and decision-support systems are being developed to analyze BP data and help clinicians tailor personalized care plans [9]. However, while these interventions are becoming more common in practice, their clinical effectiveness and sustainability across different populations remain subjects of ongoing research.

Previous reviews have examined various aspects of digital health in chronic disease management, but findings specific to hypertension are heterogeneous [10,11]. Differences in study design, population characteristics, intervention types, and outcome measures complicate the interpretation and generalization of results. Moreover, the growing number of randomized controlled trials (RCTs) published in recent years warrants an updated synthesis to capture the latest evidence and technological advances. Understanding which digital and telemedicine approaches most effectively improve BP control, treatment adherence, and lifestyle outcomes is critical for informing clinical practice and health policy.

Therefore, this systematic review aims to comprehensively evaluate evidence from RCTs assessing the effectiveness of digital health and telemedicine interventions in the management of hypertension among adults. The review focuses on key clinical and behavioral outcomes, including changes in systolic and diastolic BP, BP control rates, medication adherence, and lifestyle modifications. By synthesizing the most recent and high-quality evidence, this review seeks to identify effective digital strategies, highlight implementation challenges, and guide future research and policy efforts toward optimizing hypertension management through technology-enabled care.

## Review

Methodology

Protocol and Registration

This systematic review was conducted following the Preferred Reporting Items for Systematic Reviews and Meta-Analyses (PRISMA 2020) guidelines [12]. The review protocol was designed a priori to ensure transparency and methodological rigor. Although not registered in PROSPERO, the study design, inclusion criteria, and analytical framework were developed based on established best practices in systematic review methodology.

Eligibility Criteria

Studies were included based on the population, intervention, comparison, outcomes and study design (PICOS) framework [13], as presented in Table 1. Only RCTs were considered eligible because they represent the highest level of evidence for determining the effectiveness of interventions by minimizing selection and confounding biases. To ensure that findings reflect the latest technological developments and implementation practices, only studies published between January 2020 and October 2025 were included. Studies were excluded if they were non-randomized, observational, or qualitative in design, or if they focused on pediatric populations, pregnant women, or conditions other than hypertension. Additionally, studies not available in English or without full-text access were excluded.

**Table 1:** Eligibility criteria based on the PICOS framework. PICOS: population, intervention, comparison, outcomes and study design.

Parameter	Description
Population (P)	Adults (≥18 years) diagnosed with hypertension, irrespective of gender, ethnicity, or comorbidities.
Intervention (I)	Digital health and telemedicine interventions, including mobile health (mHealth) apps, SMS reminders, remote blood pressure monitoring, web-based counseling, teleconsultations, and AI-based decision support systems.
Comparator (C)	Usual care, standard clinical management, or non-digital interventions.
Outcomes (O)	Primary outcomes: change in systolic and diastolic blood pressure, rate of blood pressure control. Secondary outcomes: medication adherence, lifestyle modification, and adverse events.
Study design (S)	Randomized controlled trials (RCTs) published between 2020 and 2025.

Information Sources and Search Strategy

A comprehensive literature search was performed across PubMed, Scopus, Embase, Web of Science, and ClinicalTrials.gov databases. The search covered studies published between January 2020 and October 2025 to capture recent developments in digital and telemedicine interventions. The search strategy combined both Medical Subject Headings (MeSH) and free-text terms, including keywords such as “hypertension”, “digital health”, “telemedicine”, “mHealth”, “mobile application”, “blood pressure management”, and “remote monitoring”. Boolean operators (“AND” and “OR”) were used to refine search results, and reference lists of relevant articles were manually screened to identify additional studies.

Selection Process

All search results were imported into EndNote X9 (Clarivate Analytics, London, UK) for citation management and duplicate removal. Two reviewers independently screened titles and abstracts against the inclusion criteria. Full texts of potentially eligible articles were retrieved and reviewed for final inclusion. Disagreements between reviewers were resolved through discussion and, when necessary, consultation with a third reviewer. The selection process was documented using a PRISMA 2020 flow diagram to ensure transparency and reproducibility.

Data Extraction Process

A standardized data extraction form was developed and piloted prior to use. Extracted data included study characteristics (author, year, country, design, and sample size), population demographics, intervention and comparator details, duration of follow-up, and key clinical and behavioral outcomes (e.g., systolic and diastolic BP, adherence rates, and adverse events). Two reviewers independently performed the data extraction, and any discrepancies were resolved by consensus.

Risk of Bias Assessment

The Revised Cochrane Risk-of-Bias Tool for Randomized Trials (RoB 2) [14] was used to assess the methodological quality of the included studies. The domains assessed included bias arising from the randomization process, deviations from intended interventions, missing outcome data, measurement of outcomes, and selection of reported results. Each study was rated as having a low, high, or some concerns of bias. The risk-of-bias assessment was conducted independently by two reviewers, with discrepancies resolved through consensus.

Data Synthesis

Given the heterogeneity observed across studies in terms of intervention types, delivery modes, duration, and outcome measures, meta-analysis was not performed. Instead, a narrative synthesis approach was employed to qualitatively summarize the evidence. This decision ensured an accurate representation of diverse intervention strategies and prevented potential misinterpretation arising from statistically pooling heterogeneous data. The narrative synthesis focused on key trends, intervention effectiveness, and reported clinical outcomes, providing a clear understanding of the role of digital health and telemedicine in hypertension management.

Results

Study Selection Process

**Figure 1:**
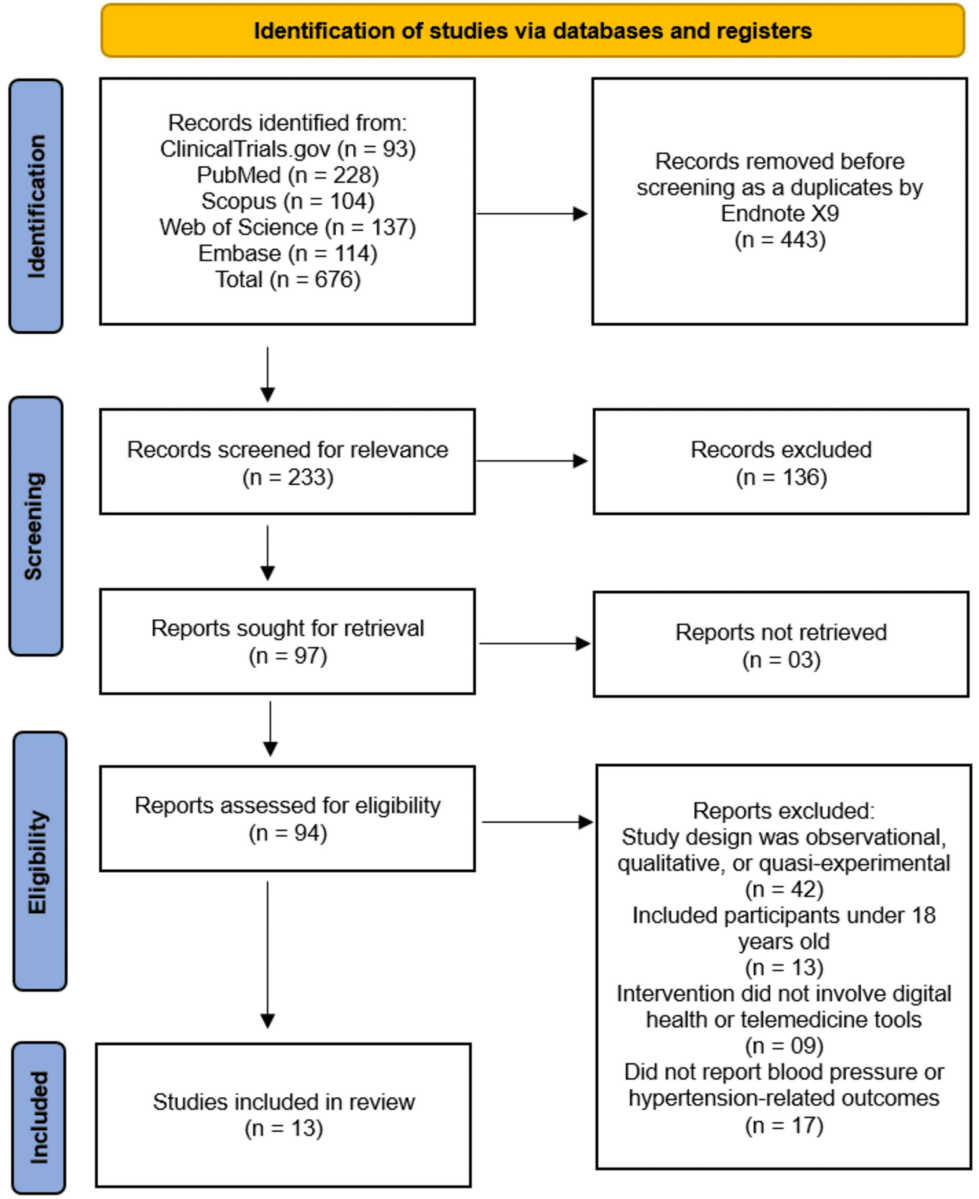
PRISMA diagram illustrating the study selection process for the systematic review.

The systematic search across five databases and registers (ClinicalTrials.gov, PubMed, Scopus, Web of Science, and Embase) initially identified 676 records. After the removal of 443 duplicate records, a total of 233 unique records were screened by title and abstract. Of these, 136 records were excluded for not meeting the broad relevance criteria. The remaining 97 reports were sought for retrieval, with 3 reports not being accessible, leaving 94 full-text articles to be assessed for eligibility. Upon detailed review, 81 reports were excluded for the following reasons: the study design was observational, qualitative, or quasi-experimental (n = 42); the included participants were under 18 years of age (n = 13); the intervention did not involve digital health or telemedicine tools (n = 9); or the study did not report BP or hypertension-related outcomes (n = 17). This rigorous process culminated in 13 studies [15-27] that met all eligibility criteria for inclusion in the systematic review (Figure 1).

Overview of Included RCTs

A total of 13 RCTs [15-27] were included in this systematic review, all published in 2020, with the exception of one study published in 2025 [15]. The characteristics of these studies are summarized in Table 2. The studies were conducted across a diverse range of countries and healthcare settings, including the USA [21,23,26], China [18,20,24], Korea [17,22], Germany [15], Canada [16], Hong Kong [19], Finland [25], and Bangladesh [27].

**Table 2:** Characteristics of included studies.

Study	Country	Study design	Sample size (n)	Population (age, gender)	Intervention type	Comparator	Duration of intervention	Follow-up	Key outcomes measured
Beger et al. (2025) [15]	Germany	Pragmatic, non-blinded RCT	606 (259 intervention, 249 control)	Adults with hypertension; intervention group mean age 55.9 ± 12.9 years; gender not specified	Manoa smartphone app integrating guideline-compliant home BP monitoring and lifestyle coaching	Standard care for hypertension	3 months	120 ± 14 days	Office systolic BP, diastolic BP, BP control, adherence to home BP monitoring
Liu et al. (2020) [16]	Canada	RCT	264	Hypertensive adults, both genders	e-Counseling for lifestyle behavior changes	Control (usual care)	Not explicitly reported	4 months, 12 months	Blood pressure (primary), exercise behaviors (daily steps), dietary behaviors (urinary sodium), FRI
Yun et al. (2020) [17]	Korea	RCT	106 (53/53)	Adults with hypertension, diabetes, or hypercholesterolemia	Smart healthing app/PC program	Educational material	3 months	12 weeks	SBP, HbA1c, LDL, % achieving targets
Sun et al. (2020) [18]	China (First Hospital of Shanxi Medical University)	RCT	120	Adults with hypertension	WeChat-guided mobile health management	Conventional management	3 months	Not explicitly stated	SBP, DBP, CPAT scores, HPSMBRS scores, BMI, urinary microalbumin, blood lipids (TC, LDL-C)
Or et al. (2020) [19]	Hong Kong	RCT, parallel-group	299 (intervention: 151, control: 148)	Diabetic and hypertensive outpatients	Tablet-based TSN app prototype	Conventional self-management	24 weeks	Baseline, 8, 12, 16, 24 weeks	Primary: hemoglobin A1c, systolic BP, diastolic BP; secondary: other indices
Gong et al. (2020) [20]	China	RCT	480	Adults with hypertension	Use of “Yan Fu” m-Health app for blood pressure management	No m-Health app (usual care)	Not specified	Not specified	Systolic and diastolic blood pressure changes, BP control rate, medication adherence
Dorsch et al. (2020) [21]	USA	RCT, open-label	50	Adults ≥ 18, M/F	LowSalt4Life app	Usual advice	8 weeks	8 weeks	Urinary sodium, dietary sodium, BP, confidence
Cho et al. (2020) [22]	Korea	3-arm RCT, single-blind	129 (41/45/43)	Adults 30-59 years old, both genders, ≥2 metabolic risk factors	App only; app + coaching	Control	24 weeks	0, 6, 12, 24 weeks	SBP, DBP, weight, body fat, waist, insulin resistance, TG, HDL
Chandler et al. (2020) [23]	USA	RCT, 2-arm, small-scale efficacy	30	Adults, mean age 45.0 years; 15 males, 15 females; 16 White, 14 Black	Breathing meditation smartphone app (Tension Tamer, TT)	Lifestyle education program delivered via smartphone (SPCTL)	12 months	12 months	Primary: change in resting SBP; secondary: change in resting DBP, adherence to TT protocol, perceived stress levels
Zhai et al. (2020) [24]	China (Xi’an City, Shaanxi Province)	Two-arm cluster RCT	384	Mean age: 68.5 ± 7.9 (intervention), 69.4 ± 9.7 (control); Female: 69%	SMS text messaging + pharmacy student–led consultations	Usual care	3 months	3 months	Systolic BP, diastolic BP, medication adherence, knowledge about hypertension, BP control rate
Tahkola et al. (2020) [25]	Finland	Cluster RCT	111	30-75 years old, M/F	Text messages + checklist	Usual care	12 months	12 months	Office SBP < 140, home SBP < 135, SBP change, meds, healthcare use, patient feedback
Schroeder et al. (2020) [26]	USA	RCT	295 (IVR-T: 148, UC: 147)	Adults, multiple races/ethnicities	IVR + texts + BP cuff	Usual care	12 months	6 and 12 months	SBP/DBP change, med adherence, visit keeping
Jahan et al. (2020) [27]	Bangladesh	RCT, open-label, parallel	420	≥35 years, M/F	Health ed. + SMS (mHealth)	Health ed. only	5 months	Not stated	Behavior change, BP, weight, salt intake, diet, physical activity, QOL

The sample sizes of the included trials varied considerably, ranging from a small-scale efficacy trial of 30 participants [23] to larger multicenter trials with over 600 participants [15]. The study populations primarily consisted of adults diagnosed with hypertension, though several studies also included individuals with comorbidities such as type 2 diabetes [19], hypercholesterolemia [17], or multiple metabolic risk factors [22]. The mean age of participants was generally middle-aged to older adults.

The digital health interventions investigated were heterogeneous in their design and delivery. A majority of the studies evaluated smartphone application-based interventions. These apps served various functions, including acting as a digital BP coach integrating guideline-compliant home BP monitoring [15], providing lifestyle and behavioral coaching [16,17,20,22], delivering mindfulness and meditation exercises [23], and offering contextual just-in-time support for dietary sodium reduction [21]. Other technological approaches included interactive voice response (IVR) systems combined with text messages [26], SMS text messaging alone or supplemented with personal consultations [24,25,27], and a tablet-based technological surrogate nursing (TSN) program [19]. The comparator in most trials was usual care, which typically consisted of conventional hypertension management without the specific digital component being tested. The duration of the interventions also varied, from short-term pilots of eight weeks [21] to longer-term programs lasting 12 months [23,25,26].

Systolic and Diastolic BP Outcomes

The impact of digital health interventions on SBP was a primary outcome in most studies, with many reporting statistically significant and clinically meaningful reductions. As detailed in Table 3, several app-based interventions demonstrated substantial SBP lowering. Beger et al. [15] reported a mean SBP reduction of -15.2 ± 14.4 mmHg using a digital BP coach. Similarly, Dorsch et al. [21] found a significantly greater SBP reduction in their intervention group compared to the control (-7.5 mmHg vs -0.7 mmHg), and Chandler et al. [23] reported a -11.6 mmHg reduction with a breathing meditation app versus -0.4 mmHg in the control group. In a three-arm trial, Cho et al. [22] observed SBP reductions across all groups, with the greatest decrease (-10.95 mmHg) in the app-only arm.

**Table 3:** Clinical and behavioral outcomes of interventions.

Study	SBP change (mean ± SD)	DBP change (mean ± SD)	BP control rate (%)	Medication adherence (%)	Lifestyle changes
Beger et al. (2025) [15]	-15.2 ± 14.4 mmHg	NR	NR	Home BP diary adherence 69.1%	Lifestyle coaching integrated
Liu et al. (2020) [16]	NR	NR	NR	NR	Daily steps ↑ at 12 months; urinary sodium ↓ in females at 12 months
Yun et al. (2020) [17]	Significant improvement; 72.7% vs 35.7% achieving SBP < 140 mmHg	NR	72.7% (intervention) vs 35.7% (control)	NR	NR
Sun et al. (2020) [18]	SBP ↓ (p < 0.001 vs baseline, p = 0.016 vs control)	DBP ↓ (p < 0.001 vs baseline, p = 0.016 vs control)	NR	NR	Improved self-management, BMI ↓, TC and LDL-C ↓, urinary microalbumin ↓
Or et al. (2020) [19]	NR	NR	NR	NR	Improved self-care suggested
Gong et al. (2020) [20]	Greater reduction than control	Greater reduction than control	Higher than control	Higher than control	NR
Dorsch et al. (2020) [21]	-7.5 mmHg vs -0.7 mmHg	NR	NR	NR	Greater reduction in dietary sodium (-1537 mg vs -515 mg by FFQ)
Cho et al. (2020) [22]	-10.95 ± 2.09/-7.29 ± 1.83/-7.19 ± 1.66	NR	NR	NR	None/self-logging/self-logging + coaching
Chandler et al. (2020) [23]	-11.6 mmHg vs -0.4 mmHg	NR	NR	Partial adherence reported	Breathing meditation vs walking
Zhai et al. (2020) [24]	134.5 ± 15.5 (intervention) vs 140.7 ± 15.2 (control)	78.2 ± 9.0 (intervention) vs 77.2 ± 10.3 (control)	98/192 (51.0%) vs 78/192 (40.6%), p = 0.08	7.4 ± 1.2 vs 7.0 ± 1.3	NR
Tahkola et al. (2020) [25]	23 vs 21 mmHg	NR	28%-36% vs 31-42%	Similar	Patients found intervention useful
Schroeder et al. (2020) [26]	0.23 mmHg	1.34 mmHg	NR	Improved (comparable to usual care)	NR
Jahan et al. (2020) [27]	SBP ↓ significantly (p = 0.04)	DBP ↓ significantly (p = 0.02)	NR	↑ adherence for salt intake and physical activity in control by 9%	Improved salt restriction, fruit and vegetable intake, physical activity, BP and weight monitoring

Other technology modalities also showed positive effects. Sun et al. [18] reported significant SBP reductions from baseline in their WeChat-guided management group, which were also significant when compared to the control group. Zhai et al. [24] observed a lower final SBP in the intervention group (134.5 mmHg) compared to the control (140.7 mmHg) after a combined SMS and consultation intervention. Jahan et al. [27] also reported a statistically significant SBP reduction in their SMS-based mHealth group in a rural Bangladeshi population.

While many studies focused on SBP, several also reported positive effects on DBP. Sun et al. [18] and Jahan et al. [27] reported significant DBP reductions in their intervention groups. Gong et al. [20] noted a "greater reduction" in DBP compared to the control, though specific values were not provided. However, some studies, including Beger et al. [15] and Dorsch et al. [21], did not report DBP outcomes.

BP Control and Medication Adherence

BP control rate, typically defined as achieving a BP below a specific threshold (e.g., <140/90 mmHg), was another key outcome. Yun et al. [17] reported a significantly higher proportion of participants achieving an SBP < 140 mmHg in the intervention group compared to the control (72.7% vs 35.7%). Gong et al. [20] similarly noted a "higher than control" BP control rate. Zhai et al. [24] found a non-significant trend toward improved control (51.0% vs 40.6%, p = 0.08), while Tahkola et al. [25] reported comparable control rates between the intervention and control groups (28%-36% vs 31%-42%).

Medication adherence was explicitly measured in several studies. Beger et al. [15] reported a 69.1% adherence to home BP diary use. Gong et al. [20] found that medication adherence was higher in their intervention group using the "Yan Fu" app. Zhai et al. [24] reported a slightly higher medication adherence score in their intervention group (7.4 vs 7.0). Schroeder et al. [26] noted improved medication adherence that was comparable to usual care, and Jahan et al. [27] reported a 9% increase in adherence to salt intake and physical activity recommendations in the control group, which only received health education.

Lifestyle and Behavioral Changes

Digital interventions demonstrated a significant impact on various lifestyle and behavioral outcomes. Improvements in dietary habits, particularly sodium reduction, were observed. Liu et al. [16] reported a decrease in urinary sodium in female participants at 12 months, while Dorsch et al. [21] found a substantially greater reduction in dietary sodium intake in their LowSalt4Life app group compared to the control (-1537 mg vs -515 mg). Jahan et al. [27] also documented improved salt restriction, along with increased fruit and vegetable intake.

Increased physical activity was another positive outcome. Liu et al. [16] reported an increase in daily steps at the 12-month follow-up. Jahan et al. [27] also noted improvements in physical activity levels. Furthermore, interventions led to enhancements in self-management and self-care capabilities. Sun et al. [18] reported improved self-management behaviors and reductions in body mass index (BMI), total cholesterol (TC), and low-density lipoprotein cholesterol (LDL-C). Or et al. [19] suggested that their TSN app improved self-care among patients with diabetes and hypertension. The interventions by Cho et al. [22] involved self-logging of health data, which is a key self-management behavior.

Risk of Bias in Included Studies

The methodological quality of the included studies was assessed using the Cochrane RoB 2 tool. The majority of the trials were judged to have a low risk of bias overall [15-19,21-26]. However, two studies raised some concerns. The trial by Gong et al. [20] was rated with "some concerns" due to issues in the randomization process and potential missing outcome data. The study by Jahan et al. [27] was judged to have a high risk of bias, primarily stemming from concerns in the randomization process, potential deviations from the intended interventions, and missing outcome data. For all other studies [15-19,21-26], the processes for randomization, adherence to interventions, handling of outcome data, measurement of outcomes, and selection of reported results were deemed sufficient to assign a low risk of bias rating (Table 4).

**Table 4:** Risk of bias assessment for included studies using the revised Cochrane risk-of-bias tool for randomized trials (RoB 2)

Study	D1: randomization process	D2: deviations from intended interventions	D3: missing outcome data	D4: measurement of the outcome	D5: selection of reported result	Overall risk of bias
Beger et al. (2025) [15]	Low	Low	Low	Low	Low	Low
Liu et al. (2020) [16]	Low	Low	Low	Low	Low	Low
Yun et al. (2020) [17]	Low	Low	Low	Low	Low	Low
Sun et al. (2020) [18]	Low	Low	Low	Low	Low	Low
Or et al. (2020) [19]	Low	Low	Low	Low	Low	Low
Gong et al. (2020) [20]	Some concerns	Low	Some concerns	Low	Some concerns	Some concerns
Dorsch et al. (2020) [21]	Low	Low	Low	Low	Low	Low
Cho et al. (2020) [22]	Low	Low	Low	Low	Low	Low
Chandler et al. (2020) [23]	Low	Low	Low	Low	Low	Low
Zhai et al. (2020) [24]	Low	Low	Low	Low	Low	Low
Tahkola et al. (2020) [25]	Low	Low	Low	Low	Low	Low
Schroeder et al. (2020) [26]	Low	Low	Low	Low	Low	Low
Jahan et al. (2020) [27]	Some concerns	Some concerns	Some concerns	Low	Low	High

Discussion

This systematic review synthesized evidence from 13 RCTs evaluating the efficacy of digital health and telemedicine interventions for hypertension management in adults. The collective findings indicate that these technologies hold substantial promise as effective tools for improving a range of clinical and behavioral outcomes. The most consistent finding across the included studies was the significant reduction in SBP achieved through diverse digital modalities. Interventions such as the digital BP coach by Beger et al. [15], which integrated guideline-compliant home monitoring and lifestyle coaching, demonstrated a substantial mean SBP reduction of -15.2 mmHg. Similarly, focused apps like the LowSalt4Life intervention by Dorsch et al. [21] and the Tension Tamer meditation app by Chandler et al. [23] resulted in clinically meaningful SBP decreases of -7.5 mmHg and -11.6 mmHg versus their respective controls. These findings are particularly compelling as they span different intervention types, from comprehensive management platforms to targeted behavioral tools, suggesting a robust effect of digital engagement on this key cardiovascular risk factor. The positive effects were not limited to app-based solutions; simpler technology such as the SMS text messaging and pharmacy student consultations employed by Zhai et al. [24] also led to a final SBP that was 6.2 mmHg lower in the intervention group. This convergence of evidence across technological complexity underscores the potential for scalable solutions even in resource-limited settings, a point further supported by the significant SBP reduction reported by Jahan et al. [27] in a rural Bangladeshi population using basic mHealth SMS.

The positive impact on DBP, while reported in fewer studies, further strengthens the case for digital interventions. Sun et al. [18] and Jahan et al. [27] documented statistically significant DBP reductions, and Gong et al. [20] noted a "greater reduction" compared to the control. The mechanisms behind these BP-lowering effects are likely multifaceted, extending beyond mere physiological monitoring to encompass enhanced self-management and behavioral modification. The outcomes related to BP control rates provide a more pragmatic measure of success, translating BP reductions into clinically relevant endpoints. The dramatic difference reported by Yun et al. [17], with 72.7% of the intervention group achieving an SBP target compared to 35.7% in the control, highlights how digital programs can effectively guide patients toward treatment goals. While not all studies showed a statistically significant improvement in control rates, as seen in the trend reported by Zhai et al. [24], the overall direction of effect supports the utility of these interventions in achieving standardized clinical benchmarks.

When contextualized within the broader literature, our findings align with and extend previous meta-analyses and reviews. For instance, a seminal meta-analysis by Nkyekyer et al. [28] on mobile health interventions for hypertension concluded that these tools significantly reduced SBP (mean difference -4.71 mmHg) and DBP compared to usual care, a finding that is corroborated and exceeded in magnitude by several studies in our review, such as Beger et al. [15] and Chandler et al. [23]. The diversity of effective platforms in our review, from smartphone apps to SMS, echoes the conclusions of a network meta-analysis by Li et al. [29], which found that while all digital interventions were generally effective, those incorporating multiple functions like monitoring, feedback, and education tended to have larger effect sizes. This is perfectly exemplified in our review by the multi-component intervention of Sun et al. [18], which led to improvements not only in BP but also in self-management scores, BMI, and lipid profiles. Our results regarding the efficacy of text messaging support the work of Zangger et al. [30], who specifically focused on SMS interventions and found a significant, though modest, reduction in SBP. The findings from Zhai et al. [24] and Jahan et al. [27] in our review fall within this spectrum, demonstrating that even low-tech solutions can yield meaningful benefits, especially in populations with limited access to smartphones or broadband internet.

Beyond clinical metrics, a critical strength of digital interventions illuminated by this review is their capacity to foster positive lifestyle and behavioral changes, which are the cornerstone of long-term hypertension control. The significant reduction in dietary sodium intake reported by Dorsch et al. [21] (-1537 mg vs -515 mg) is a standout finding, as sodium restriction is one of the most effective non-pharmacological strategies for BP management. This aligns with a systematic review by Li et al. [29] on digital health for salt reduction, which confirmed the efficacy of technology-based interventions in lowering sodium intake, primarily through self-monitoring and tailored feedback. Similarly, the increase in daily steps reported by Liu et al. [16] and the improvements in physical activity and fruit and vegetable intake documented by Jahan et al. [27] demonstrate the ability of these platforms to address multiple cardiovascular risk factors simultaneously. This holistic approach is crucial, as emphasized in a comprehensive review by Burke et al. [31] on the role of digital health in cardiovascular disease prevention, which argued that the greatest potential lies in multifactorial interventions that target diet, activity, and medication adherence concurrently. The improvements in self-management and self-care reported by Sun et al. [18] and suggested by Or et al. [19] point to a fundamental mechanism of action: digital tools empower patients by providing them with the data, knowledge, and feedback loops necessary to take an active role in their own health management. This is consistent with the concept of the "activated patient" described in the Chronic Care Model, where informed, engaged patients are more likely to achieve positive health outcomes.

Medication adherence, a perennial challenge in chronic disease management, was also positively influenced by several digital interventions. The higher medication adherence reported by Gong et al. [20] and the improved adherence scores in the study by Zhai et al. [24], though modest, are clinically significant. These findings are supported by a large meta-analysis by Nieuwlaat et al. [32], which found that interventions including reminders, self-monitoring, and pharmacist support, elements present in many of the apps and SMS systems we reviewed, were among the most effective at improving adherence. The improved adherence to non-pharmacological recommendations, such as salt intake and physical activity in the study by Jahan et al. [27], further underscores the role of digital tools in supporting a broad range of treatment adherence behaviors.

The generally low risk of bias in the majority of the included studies [15-19,21-26] lends considerable weight to these findings. The use of randomized controlled designs across diverse geographical and clinical settings enhances the generalizability and robustness of the evidence base. However, the heterogeneity of the interventions, ranging from breathing meditation apps to comprehensive surrogate nursing systems, makes it challenging to pinpoint a single "best" technological approach. This heterogeneity reflects a rapidly evolving field where innovation is occurring across multiple fronts. Rather than being a weakness, this diversity suggests that the optimal digital intervention may be context-dependent, tailored to specific patient populations, resources, and healthcare systems. For example, the highly sophisticated tablet-based TSN app by Or et al. [19] may be ideal for complex patients with comorbidities in a well-resourced setting, while the simple SMS-based intervention by Jahan et al. [27] provides a viable and effective model for large-scale public health initiatives in low- and middle-income countries.

Limitations

Several limitations of this review and the included studies must be acknowledged. First, the high degree of clinical and methodological heterogeneity among the interventions precluded a meta-analysis, limiting our ability to provide pooled effect estimates. Second, while the risk of bias was mostly low, some concerns remain, particularly for the studies by Gong et al. [20] and Jahan et al. [27], which may affect the reliability of their findings. Third, the relatively short duration of many trials (e.g., three months in several studies) leaves questions about the long-term sustainability of the observed benefits. Fourth, reporting was often incomplete; many studies did not report diastolic BP outcomes [15,21,23] or provided only qualitative descriptions of improvements [19,20], which limits the depth of our analysis. Furthermore, the digital divide remains a critical issue. The effectiveness of these interventions is contingent on access to technology and digital literacy, which may exacerbate health inequalities if not deliberately addressed. Finally, the rapid pace of technological advancement means that the specific apps and platforms evaluated may quickly become obsolete, though the underlying principles of remote monitoring, personalized feedback, and behavioral coaching are likely to remain relevant.

## Conclusions

Digital health and telemedicine interventions are effective adjuncts to standard care for managing hypertension in adults. These technologies consistently lead to significant reductions in systolic BP, improve rates of BP control, and promote beneficial lifestyle changes and medication adherence. The effects are demonstrated across a spectrum of technologies, from complex smartphone applications to simple text messaging, indicating a versatile and scalable approach to a major public health challenge. Future research should focus on implementing longer-term trials to assess sustainability, conducting head-to-head comparisons of different technological components to identify the most active ingredients, and proactively designing strategies to ensure equitable access across diverse socioeconomic and demographic groups. Integrating these evidence-based digital tools into structured hypertension management programs has the clear potential to alleviate the global burden of cardiovascular disease.
